# Comparative Evaluation of Corticosterone Administration, Chronic Restraint Stress, and Their Combination for Depression-like Behavioral and Molecular Alterations in Mice: A Multi-Domain Assessment

**DOI:** 10.3390/ijms27146277

**Published:** 2026-07-14

**Authors:** Chang-Ho Shin, Sun-Min Jin, Da-Jung Hwang, Myeong-Hyun Nam, Hee-Deok Yun, Yuna Kim, Ju-Yeong Lee, Young-Kwon Seo

**Affiliations:** 1 Department of AI Convergence Biomedical Engineering, Dongguk University, Goyang-si 10326, Republic of Korea; changhoshin84@gmail.com; 2 AriBio Co., Ltd., 56, Dongpangyo-ro, Bundang-gu, Seongnam-si 13535, Republic of Korea; smjin@aribio.com (S.-M.J.); dahwang@aribio.com (D.-J.H.); 3 Department of Biomedical Engineering, Dongguk University, Goyang-si 10326, Republic of Korea; iis05047@naver.com (M.-H.N.); drengon1538@gmail.com (H.-D.Y.); amy0309kr@naver.com (Y.K.); leejulialee2002@gmail.com (J.-Y.L.)

**Keywords:** depression, corticosterone, chronic restraint stress, mouse model, hippocampus, neuroinflammation, BDNF, serotonin, LC-MS/MS, adenosine, dopamine, ERK/CREB signaling

## Abstract

Major depressive disorder (MDD) involves dysregulation of the hypothalamic–pituitary–adrenal axis, neuroinflammation, serotonergic dysfunction, and impaired neurotrophic signaling. Whether combining corticosterone (CORT) with chronic restraint stress (CRS) produces a more comprehensive depression-like phenotype than either model alone remains unexplored. Male C57BL/6N mice were assigned to four groups—Sham (*n* = 7), CORT (20 mg/kg s.c.; *n* = 7), CRS (3 h/day; *n* = 7), and CORT + CRS (C+C; *n* = 8)—and evaluated by behavioral tests, hippocampal qRT-PCR, Western blot, LC-MS/MS metabolomics, and immunohistochemistry. All experimental groups showed elevated immobility in tail suspension and forced swim tests without inter-group differences. Two-way ANOVA revealed a behavioral–molecular dissociation: FST immobility showed CORT and CRS main effects without a significant interaction, whereas hippocampal LC-MS/MS analytes exhibited strong CORT × CRS interactions. The C+C group showed the strongest 5-HT1A receptor (HTR1A) upregulation, the greatest reductions in Trk-b and DCX, and unique decreases in serum dopamine, glutamine, and adenosine. Representative Western blot densitometry indicated the largest p-ERK/ERK reduction in C+C (54%), while p-CREB/CREB was most suppressed in CRS (52%). BDNF and NeuN proteins were lowest in C+C (68% and 61% reductions). Combined CORT + CRS produces the most comprehensive depression-related molecular profile, integrating multiple pathological domains.

## 1. Introduction

Major depressive disorder (MDD) affects over 300 million people worldwide and remains a leading cause of disability across all age groups [1]. Despite decades of pharmacological and psychotherapeutic advances, treatment-resistant depression affects approximately 30% of patients, highlighting the persistent need for improved preclinical models that faithfully recapitulate the complex pathophysiology of human depression [2,3]. The heterogeneous nature of MDD, involving dysregulated hypothalamic–pituitary–adrenal (HPA) axis function, chronic neuroinflammation, serotonergic imbalance, impaired neurotrophic signaling, and glutamatergic dysfunction, demands animal models that capture multiple pathological dimensions simultaneously [2,4].

Among the most established rodent depression models, chronic corticosterone (CORT) administration and chronic restraint stress (CRS) represent fundamentally different approaches to modeling the disorder [5,6,7]. Exogenous CORT injection directly simulates the sustained hypercortisolemia observed in a substantial proportion of depressed patients, bypassing higher-order stress processing to act directly on glucocorticoid receptors in the hippocampus, prefrontal cortex, and amygdala [7]. Zhao et al. [8] established the foundational protocol of repeated subcutaneous CORT injection at 20 mg/kg, demonstrating time-dependent increases in despair-related behaviors and hippocampal molecular alterations. Subsequent studies have confirmed that chronic CORT administration reduces hippocampal BDNF expression through hyperactive neuronal autophagy [9], suppresses adult neurogenesis, and induces behavioral alterations that are sensitive to housing conditions and strain background [10,11]. Notably, Sturm et al. [12] demonstrated that chronic CORT treatment in C57BL/6N mice produces differential behavioral outcomes compared to C57BL/6J mice, highlighting the importance of substrain selection in CORT-based depression models. Furthermore, Jung et al. [13] showed that chronic CORT treatment alters hippocampal reelin expression alongside emotional behavioral changes, linking glucocorticoid-induced synaptic remodeling to depression-like phenotypes.

In contrast, CRS engages a naturalistic, multi-system stress response involving not only HPA axis activation but also sympathoadrenal catecholamine release, autonomic nervous system activation, and psychological distress processing [14,15]. The behavioral and molecular consequences of CRS are critically dependent on the duration and intensity of restraint exposure. Kim and Han [16] demonstrated that a single 24-h restraint episode is sufficient to induce long-term depressive-like phenotypes in mice persisting for at least two weeks, while Jeon et al. [17] showed that varying CRS periods (1, 2, or 3 weeks of daily 4-h restraint) produce differential effects on anxiety- and depressive-like behaviors as well as tryptophan–kynurenine metabolism along the brain–gut axis. Son et al. [18] further documented that restraint stress-induced depressive phenotypes vary significantly even among C57BL/6N mice derived from different commercial sources, underscoring the critical role of environmental and vendor-specific factors. Importantly, Dimitrov et al. [19] demonstrated that CRS activates the HPA axis and suppresses feeding behavior through PACAPergic neurotransmission, revealing that restraint stress engages specific neuropeptide signaling cascades beyond the classical glucocorticoid pathway.

Critically, electrophysiological evidence demonstrates that these two models engage fundamentally distinct neurobiological mechanisms. Bąk et al. [20] showed that repeated CORT administration decreased GABAergic inhibitory transmission in dorsal raphe nucleus neurons without affecting neuronal excitability, while restraint stress decreased neuronal excitability without affecting inhibitory transmission, and that the 5-HT7 receptor antagonist SB 269970 differentially modulated these effects. Furthermore, behavioral dissociation studies have revealed that CORT produces depression-like behavior without anxiety, whereas CRS produces anxiety without depression in some paradigms [21], while others show that CRS increases aggressive behavior alongside depression [22]. These findings suggest that combining the two approaches may engage complementary pathways to generate a more complete depression phenotype.

However, the systematic comparison of CORT, CRS, and their combination using comprehensive multi-domain assessment remains remarkably sparse. Only one prior study has combined CORT injection with CRS: Ngoupaye et al. [23] reported that the combined paradigm produced enhanced behavioral despair and early cognitive deficits, but their study lacked molecular or histological characterization. Recent models have explored combining CRS with other stressors—for example, Li et al. [24] developed a combined CRS and lipopolysaccharide (LPS) model—but no study has assessed the CORT+CRS combination across behavioral, inflammatory, serotonergic, neurotrophic, metabolomic, and histological domains simultaneously.

The primary aim of this study is to produce a multi-domain molecular and histological comparison of CORT, CRS, and their combination, rather than a full behavioral validation of a depression model. Accordingly, this study addresses the existing research gap by systematically comparing four experimental groups—Sham, CORT (20 mg/kg), CRS (3 h/day), and combined CORT + CRS (C+C)—across an integrated assessment battery comprising three behavioral tests, nine qRT-PCR targets in the hippocampus, Western blot protein quantification (Iba1, BDNF, NeuN, ERK/p-ERK, CREB/p-CREB), LC-MS/MS metabolomics in both hippocampal tissue and serum, and four immunohistochemical markers in the hippocampus and thalamus. We hypothesized that the combined C+C paradigm would produce a more robust and comprehensive depression-related molecular phenotype than either CORT or CRS alone, even in the absence of synergistic behavioral effects, and that the pattern of molecular changes would reveal the domain-specific contributions of each model component.

## 2. Results

### 2.1. Behavioral Changes Following Experimental Stress Exposure

#### 2.1.1. CORT Selectively Impairs Open Field Exploration

**Figure 1:**
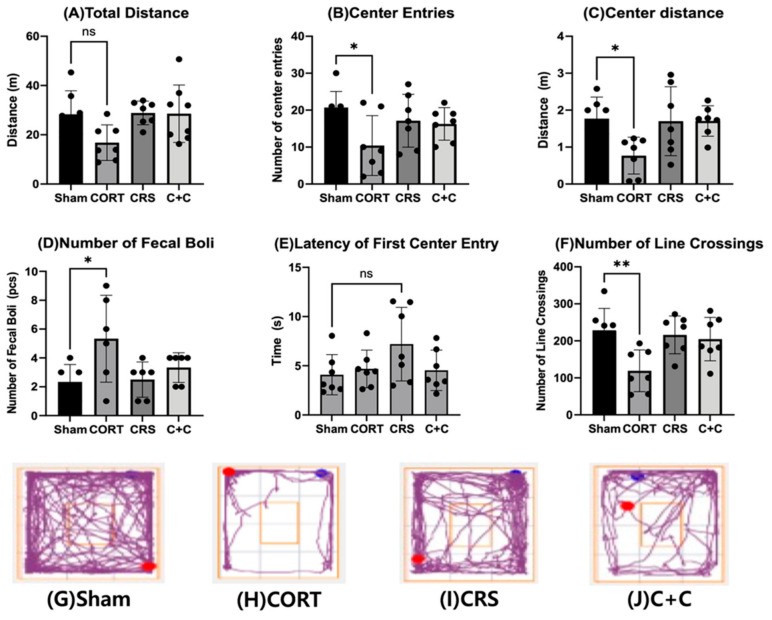
Open field test (OFT) outcomes: (**A**) total distance traveled; (**B**) number of center zone entries; (**C**) center zone distance; (**D**) number of fecal boli; (**E**) latency to first center entry; (**F**) number of line crossings; (**G**–**J**) representative movement trajectories for each group. CORT administration selectively reduced exploratory behavior; CRS and C+C groups showed no significant OFT alterations. Data represent mean ± SEM (*n* = 7–8 per group). * *p* < 0.05, ** *p* < 0.01 vs. Sham (one-way ANOVA with Tukey’s post hoc test). Full two-way ANOVA (CORT × CRS factorial) results, including F statistics, degrees of freedom, and interaction terms, are provided in the Supplementary Statistics Tables. ns, not significant.

OFT results revealed selective effects of CORT administration on exploratory behavior (Figure 1). Total distance traveled did not differ significantly across the four groups (*p* > 0.05). However, the CORT group showed significant decreases in center zone entries (*p* < 0.05 vs. Sham) and center zone distance (*p* < 0.05 vs. Sham), suggesting reduced exploratory motivation or increased anxiety-like behavior specific to the pharmacological model. The CORT group also showed a significant increase in fecal boli count (*p* < 0.05 vs. Sham), an indicator of autonomic stress response. Line crossings were significantly decreased in the CORT group (*p* < 0.01 vs. Sham). Notably, neither the CRS nor the C+C group showed significant alterations in any OFT parameter compared to controls, indicating that CRS exposure did not induce detectable changes in open field behavior and that CORT-driven behavioral effects were attenuated in the combined paradigm. This finding aligns with previous reports that CRS-experienced mice may exhibit relative boldness or habituation-related locomotor normalization in novel environments.

#### 2.1.2. Stress Exposure Increases Immobility Without Synergistic Effects in the C+C Group

**Figure 2:**
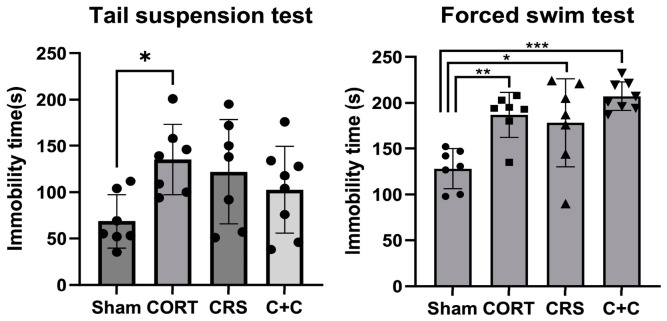
Tail suspension test (TST) and forced swim test (FST) results. Immobility time was elevated in all experimental groups compared to Sham, reaching statistical significance in TST for CORT only and in FST for all groups. No significant differences were observed among experimental groups. Data represent mean ± SEM. * *p* < 0.05, ** *p* < 0.01, *** *p* < 0.001 vs. Sham.

In the TST, immobility time was increased across all experimental groups compared to controls, although only the CORT group reached statistical significance (*p* < 0.05; Figure 2). The FST revealed more robust effects, with all experimental groups showing significantly increased immobility time compared to controls (CORT: *p* < 0.01; CRS: *p* < 0.05; C+C: *p* < 0.001). However, no statistically significant differences were observed among the three experimental groups in either TST or FST, indicating that the C+C combined paradigm did not produce synergistic effects on behavioral despair measures.

### 2.2. Hippocampal qRT-PCR Analysis

#### 2.2.1. Pro-Inflammatory Cytokine Gene Expression Is Upregulated Across Experimental Groups

**Figure 3:**
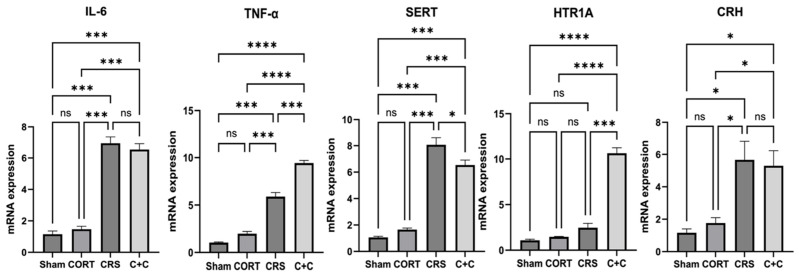
Hippocampal qRT-PCR analysis of depression-related gene expression (mRNA levels). Inflammatory markers (IL-6, TNF-α), serotonergic markers (SERT, HTR1A), stress hormone (CRH), and neurotrophic factors (BDNF, NGF, Trk-b, DCX) were assessed across all four groups. CRS-containing groups showed enhanced inflammatory and serotonergic changes; C+C exhibited the most pronounced HTR1A upregulation and Trk-b/DCX reduction. Data represent mean ± SEM. * *p* < 0.05, *** *p* < 0.001, **** *p* < 0.0001. ns, not significant.

Hippocampal gene expression of both pro-inflammatory cytokines (IL-6 and TNF-α mRNA) was significantly upregulated in all experimental groups compared to controls (Figure 3). For IL-6, CRS and C+C groups showed markedly greater elevation than CORT (CRS vs. Sham: *p* < 0.001; C+C vs. Sham: *p* < 0.001; CORT vs. Sham: *p* < 0.001). TNF-α followed a similar pattern, with CRS-containing groups (CRS and C+C) showing greater inflammatory activation than CORT alone. The C+C group showed a trend toward the highest TNF-α expression, consistent with additive inflammatory effects of combined treatment.

#### 2.2.2. Serotonergic Dysregulation Is Most Pronounced in the C+C Group

SERT mRNA expression was significantly increased in all experimental groups compared to controls (all *p* < 0.001), indicating elevated serotonin reuptake capacity across all depression models (Figure 3). CRS-containing groups showed greater SERT elevation than CORT alone. HTR1A (the inhibitory serotonin autoreceptor) was dramatically upregulated specifically in the C+C group (*p* < 0.0001 vs. Sham), with significantly higher expression than both the CORT and CRS groups (C+C vs. CORT: *p* < 0.001; C+C vs. CRS: *p* < 0.001). This represents one of the most pronounced synergistic molecular effects observed in this study.

#### 2.2.3. CRH Expression Is Preferentially Increased in CRS-Containing Groups

CRH mRNA expression was elevated in all experimental groups, with CRS-containing groups showing greater CRH upregulation than CORT alone (Figure 3). Statistical significance was reached for CRS vs. Sham (*p* < 0.05) and C+C vs. Sham (*p* < 0.05), while CORT vs. Sham showed a non-significant trend. Inter-group comparisons revealed that CRS was more effective than CORT at inducing CRH expression, consistent with endogenous HPA axis activation by physical stress versus expected negative feedback with exogenous glucocorticoid administration.

#### 2.2.4. Neurotrophic and Neurogenesis-Related Markers Are Suppressed Across Experimental Groups

All four neurotrophic markers were significantly decreased in experimental groups compared to controls (Figure 3). BDNF mRNA was reduced in CORT (*p* < 0.001), CRS (*p* < 0.001), and C+C (*p* < 0.001) groups, with no significant inter-group differences, consistent with the neurotrophic hypothesis of depression and evidence that diverse interventions targeting BDNF/TrkB signaling (e.g., kefir peptides, curcumin nanoparticles) can reverse this deficit. NGF showed significant reductions in CORT (*p* < 0.05) and C+C (*p* < 0.05) groups. Trk-b, the BDNF receptor, was significantly reduced in all experimental groups (all *p* < 0.05 vs. Sham), with the C+C group showing a trend toward the largest decrease. DCX, an immature neuron marker reflecting adult hippocampal neurogenesis, was significantly reduced in CORT (*p* < 0.01) and C+C (*p* < 0.01) groups, with the C+C group showing the most pronounced effect. These results demonstrate that the combined paradigm was particularly effective at disrupting the neurotrophic signaling cascade.

### 2.3. Hippocampal LC-MS/MS Analysis Shows Uniform Neurotransmitter Elevation Across Experimental Groups

**Figure 4:**
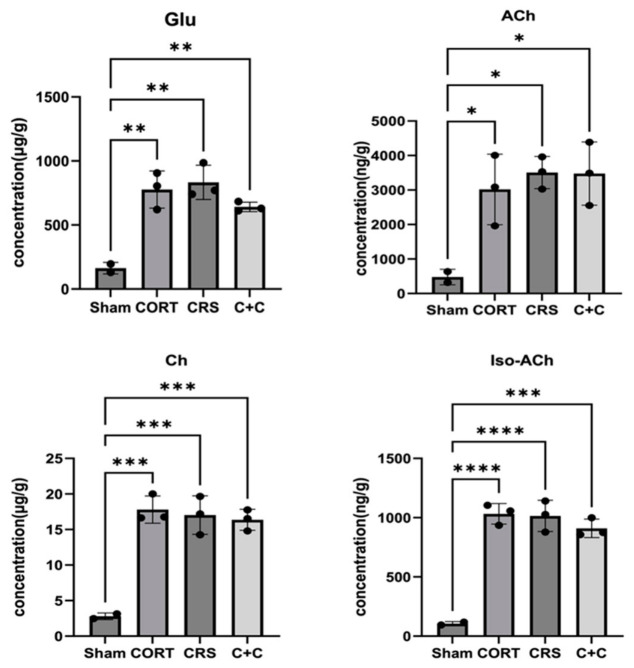
Hippocampal LC-MS/MS analysis. Concentrations of glutamate (Glu), acetylcholine (ACh), choline (Ch), and isoacetylcholine (Iso-ACh) in hippocampal tissue. All analytes were uniformly elevated across experimental groups without significant inter-group differences. Data represent mean ± SEM. * *p* < 0.05, ** *p* < 0.01, *** *p* < 0.001, **** *p* < 0.0001 vs. Sham.

LC-MS/MS quantification of hippocampal neurotransmitters and metabolites revealed uniform elevation across all experimental groups compared to controls (Figure 4). Glutamate (Glu) concentrations were significantly increased in all groups (all *p* < 0.01), consistent with reports that excitotoxic glutamatergic dysregulation and glutamate-glutamine cycle disruption are common metabolomic features across multiple depression models. Acetylcholine (ACh), choline (Ch), and isoacetylcholine (Iso-ACh) were similarly elevated across experimental groups. No significant differences were observed among the three experimental groups for any hippocampal analyte, indicating that CORT, CRS, and their combination produced equivalent effects on hippocampal cholinergic and glutamatergic neurotransmission.

### 2.4. Serum LC-MS/MS Analysis Identifies C+C-Specific Metabolic Alterations

**Figure 5:**
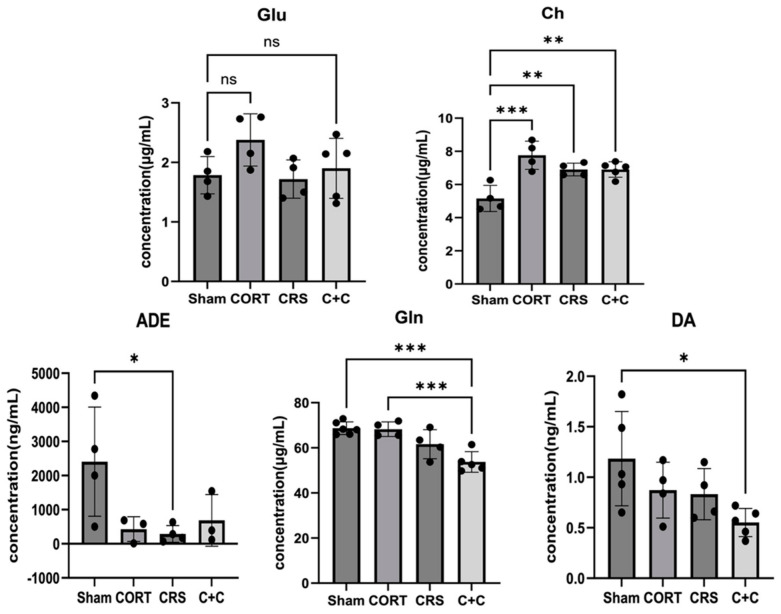
Serum LC-MS/MS analysis. Concentrations of glutamate (Glu), choline (Ch), adenosine (ADE), glutamine (Gln), and dopamine (DA) in serum. The C+C group showed unique reductions in adenosine, glutamine, and dopamine not observed in CORT or CRS alone. Data represent mean ± SEM. * *p* < 0.05, ** *p* < 0.01, *** *p* < 0.001 vs. Sham. ns, not significant.

Serum metabolite profiles revealed differential sensitivity to the combined paradigm (Figure 5). Serum glutamate did not differ significantly between groups, but serum choline was significantly elevated in all experimental groups (all *p* < 0.01 vs. Sham). Notably, the C+C group showed unique alterations in three key biomarkers: serum glutamine was significantly reduced (*p* < 0.001 vs. Sham), reflecting metabolic imbalance in the glutamate–glutamine cycle; serum dopamine was significantly reduced specifically in the C+C group (*p* < 0.05 vs. Sham), suggesting combined paradigm-specific impairment of dopaminergic transmission; and serum adenosine was also significantly decreased in the C+C group (*p* < 0.05 vs. Sham). The reduction in adenosine is particularly noteworthy given evidence that adenosine signaling modulates depressive behavior through A1 and A2A receptors, that exogenous adenosine administration produces antidepressant-like effects in mice, and that decreased adenosine deaminase activity has been reported in depressed patients. The specificity of these serum changes to the C+C group suggests they may serve as peripheral biomarker signatures distinguishing the combined HPA-stress depression phenotype.

### 2.5. Immunohistochemistry Reveals Neuroinflammatory and Neurodegenerative Changes

#### 2.5.1. CRS Produces the Strongest Microglial Activation

**Figure 6:**
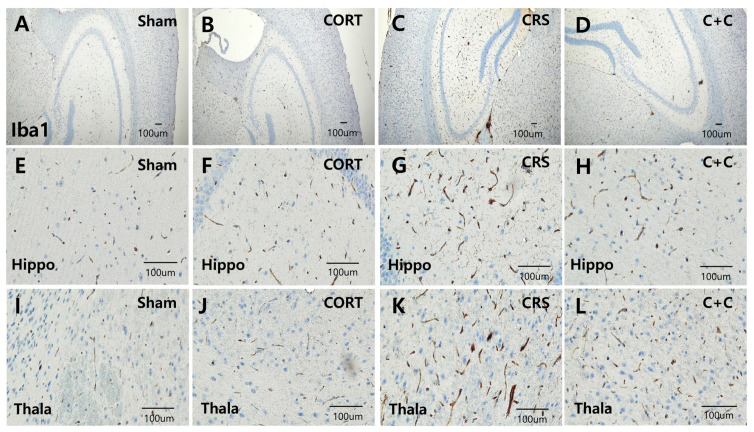
Iba1 immunohistochemistry in hippocampus and thalamus. Representative images at 40× (**A**–**D**) and 200× magnification in hippocampus (**E**–**H**) and thalamus (**I**–**L**). The CRS group showed the most prominent microglial activation. Scale bars: 100 μm.

Iba1 immunostaining in both the hippocampus and thalamus revealed the most prominent microglial activation in the CRS group (Figure 6). CRS-treated mice showed increased Iba1-positive cell density along with morphological features of activated microglia (cellular hypertrophy, thickened processes) compared to the ramified resting morphology in controls. The CORT group showed modest increases, while the C+C group exhibited intermediate activation. These findings suggest that physical restraint stress is a more potent activator of microglial responses than exogenous glucocorticoid administration, consistent with evidence that stress-induced neuroinflammation is mediated through NLRP1 inflammasome activation, FKBP51-dependent regulation of hippocampal GAD65 expression, and IL-17 signaling pathways.

#### 2.5.2. M1 Microglial Polarization Increases Across Experimental Groups

**Figure 7:**
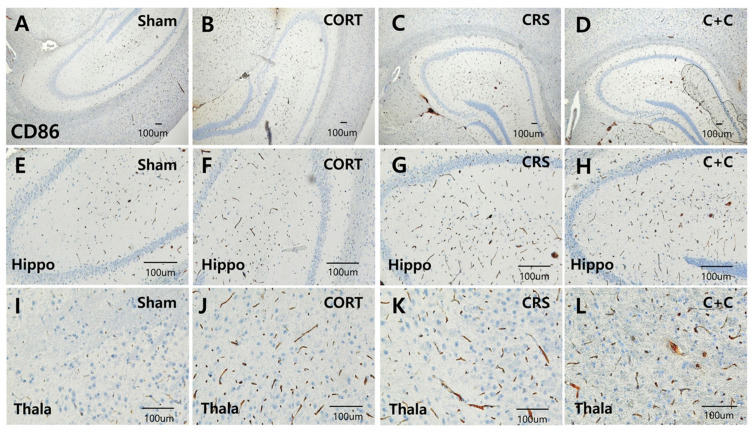
CD86 immunohistochemistry in hippocampus and thalamus. Representative images at 40× (**A**–**D**) and 200× magnification in hippocampus (**E**–**H**) and thalamus (**I**–**L**). M1 microglial polarization was increased across all experimental groups with minimal inter-group differences. Scale bars: 100 μm.

CD86 immunoreactivity, indicating pro-inflammatory M1 microglial polarization, was increased in all experimental groups compared to controls in both the hippocampus and thalamus (Figure 7). Differences among experimental groups were minimal, suggesting that both CORT and CRS independently promote M1 polarization to similar degrees without additive effects, consistent with convergent inflammatory signaling through TLR4/NF-κB pathways.

#### 2.5.3. C+C Produces the Greatest Reduction in NeuN Expression

**Figure 8:**
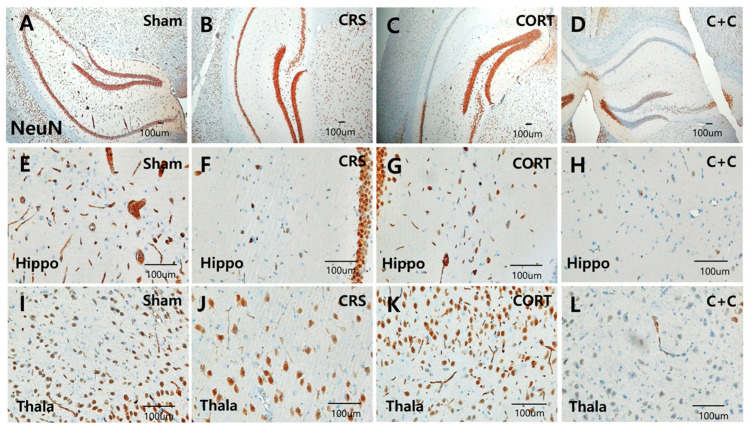
NeuN immunohistochemistry in the hippocampus and thalamus. (**A**–**D**) Hippocampus at 40× magnification; (**E**–**H**) hippocampus at 200× magnification; (**I**–**L**) thalamus at 200× magnification. NeuN staining showed a selective reduction in neuronal immunoreactivity in the CORT + CRS group. Scale bars: 100 μm.

NeuN immunostaining, reflecting mature neuronal density, showed pronounced reductions specifically in the C+C group (Figure 8). NeuN-positive cell density was decreased in the hippocampal CA1 and dentate gyrus subregions and in thalamic regions of C+C mice compared to all other groups. The CRS and CORT groups showed modest but less pronounced reductions. This finding indicates that the combined paradigm uniquely promotes neuronal loss or degeneration beyond what either treatment achieves individually, presumably through convergent mechanisms of glucocorticoid-induced autophagy and stress-mediated excitotoxicity.

#### 2.5.4. BDNF Expression Is Reduced Across Experimental Groups

**Figure 9:**
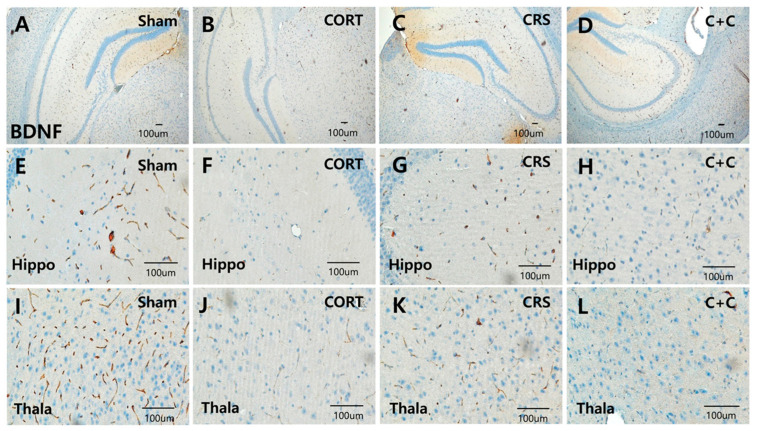
BDNF immunohistochemistry in the hippocampus and thalamus. (**A**–**D**) Hippocampus at 40× magnification; (**E**–**H**) hippocampus at 200× magnification; (**I**–**L**) thalamus at 200× magnification. BDNF immunoreactivity was decreased across all experimental groups, with the greatest reductions in the CORT and CORT + CRS groups. Scale bars: 100 μm.

BDNF protein expression assessed by immunohistochemistry was decreased in all experimental groups in both the hippocampus and thalamus (Figure 9), consistent with the qRT-PCR results. The most pronounced reductions were observed in the CORT and C+C groups, with the CRS group showing intermediate effects. The reduction in thalamus was particularly notable, with near-complete loss of BDNF immunoreactivity in the C+C group.

### 2.6. Western Blot AnalysisWestern Blot Analysis of Protein Expression and Signaling Pathways

**Figure 10:**
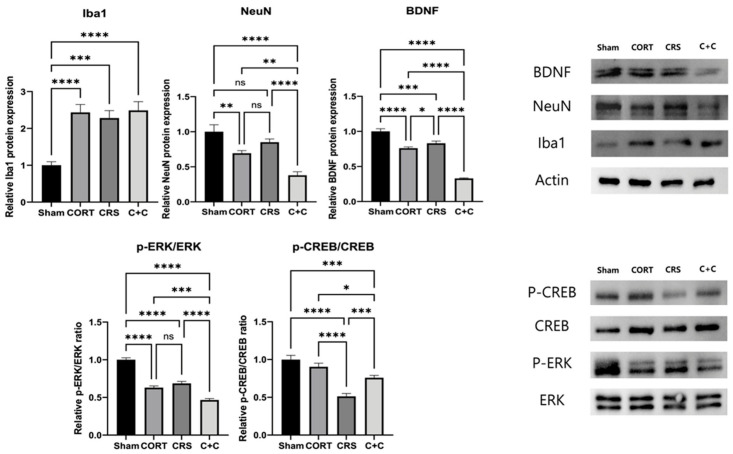
Western blot analysis of hippocampal protein expression. (A) Representative immunoblots for Iba1, BDNF, NeuN, ERK, p-ERK, CREB, and p-CREB with β-actin as loading control. Note: y-axes represent relative protein expression (fold change vs. Sham). Bars represent representative ImageJ 1.54r densitometric quantification (*n* = 1 representative lane per group). See Section 3.7 (Limitations) and Methods 4.5 for context. Lanes from left to right: Sham, CORT, CRS, C+C. (B–H) Densitometric quantification normalized to β-actin. Iba1 protein was elevated comparably across all experimental groups (CORT, 2.29-fold; CRS, 2.13-fold; C+C, 2.35-fold vs. Sham). BDNF and NeuN were reduced across experimental groups, most prominently in C+C (BDNF: 68% reduction; NeuN: 61% reduction). Total ERK and CREB remained relatively stable, while the p-ERK/ERK ratio was most strongly reduced in C+C (54%) and the p-CREB/CREB ratio was most strongly reduced in CRS (52%). Data represent mean ± SEM. * *p* < 0.05, ** *p* < 0.01, *** *p* < 0.001, **** *p* < 0.0001 vs. Sham. ns, not significant.

Representative Western blot analysis of hippocampal protein extracts was performed to confirm transcriptional findings at the protein level and to assess intracellular signaling pathway activity (Figure 10). Representative immunoblots for Iba1, BDNF, NeuN, ERK, p-ERK, CREB, and p-CREB are shown alongside densitometric quantification normalized to β-actin.

#### 2.6.1. Iba1 Expression Is Elevated Across Experimental Groups

Iba1 protein expression was substantially elevated in all three experimental groups compared to Sham, with comparable fold-increases in CORT (2.29-fold), CRS (2.13-fold), and C+C (2.35-fold) groups. No significant differences were observed among the experimental groups, indicating that all three depression-inducing paradigms produced equivalent microglial activation at the protein level. This protein-level finding is consistent with the qRT-PCR results showing IL-6 and TNF-α elevation across all experimental groups and with the Iba1 immunohistochemistry data (Figure 6).

#### 2.6.2. BDNF and NeuN Expression Are Reduced Across Experimental Groups

BDNF protein expression was reduced across all experimental groups compared to Sham, consistent with the qRT-PCR and IHC findings. The CORT and C+C groups exhibited the most pronounced reductions. NeuN protein levels were similarly decreased, with the C+C group showing the greatest reduction, providing protein-level confirmation of the C+C-specific neuronal loss observed by immunohistochemistry (Figure 8).

#### 2.6.3. C+C Most Strongly Suppresses ERK Activation, Whereas CRS Preferentially Reduces CREB Phosphorylation

Total ERK protein expression remained relatively stable across all groups (Sham, 1.00; CORT, 0.98; CRS, 0.82; C+C, 0.87 relative to Sham), while phosphorylated ERK (p-ERK) was markedly reduced in all experimental groups, indicating impaired ERK activation. The p-ERK/ERK ratio was decreased in all experimental groups, with the C+C group showing the most substantial reduction (CORT, 0.62-fold; CRS, 0.68-fold; C+C, 0.46-fold relative to Sham). In contrast, the p-CREB/CREB ratio displayed a different pattern: while total CREB levels showed minor variation across groups, the p-CREB/CREB ratio was most strongly reduced in the CRS group (0.48-fold vs. Sham), with milder reductions in CORT (0.86-fold) and C+C (0.74-fold) groups. This dissociation between p-ERK and p-CREB regulation suggests that CRS preferentially suppresses CREB-mediated transcription independently of ERK activation, possibly through alternative kinase pathways such as CaMKII signaling, whereas the combined paradigm (C+C) most severely impairs ERK phosphorylation. Collectively, these findings indicate that the ERK–CREB signaling cascade—a critical pathway linking extracellular neurotrophic signals to nuclear transcription of survival and plasticity genes including BDNF—is impaired in all depression models through partially distinct mechanisms, with C+C producing the most severe disruption at the ERK level and CRS at the CREB level. The convergent suppression of both p-ERK and the BDNF/Trk-b axis in the C+C group provides a mechanistic link to the downstream neuronal degeneration (reduced NeuN) observed specifically in this group.

## 3. Discussion

This study represents the first comprehensive multi-domain comparison of exogenous corticosterone administration, chronic restraint stress, and their combination in mice. By integrating behavioral, molecular, metabolomic, and histological assessments, we demonstrate that the combined CORT + CRS paradigm produces the most comprehensive depression-related molecular phenotype, despite the absence of behavioral synergy. These findings have important implications for preclinical depression model selection and underscore the critical importance of multi-domain assessment in model validation.

### 3.1. Behavioral–Molecular Dissociation: Implications for Depression Model Assessment

The most striking finding of this study is the marked dissociation between behavioral and molecular outcomes. While all experimental groups showed increased immobility in TST and FST compared to controls, no synergistic behavioral effects were observed in the C+C group. In contrast, molecular analyses revealed clear C+C superiority across multiple domains, including HTR1A upregulation, serum dopamine reduction, and NeuN loss. This dissociation is consistent with the growing recognition that behavioral tests like the FST reflect coping strategies rather than depression-like states [25,26]. Commons et al. [27] demonstrated that FST immobility responds to numerous non-antidepressant compounds, weakening its specificity as a depression measure, and meta-analytic evidence indicates highly heterogeneous effect sizes across studies [28,29]. Our data support the interpretation that behavioral despair responses plateau once a threshold level of stress exposure is reached, while underlying molecular pathology continues to deepen with combined treatment. This interpretation is directly supported by formal two-way ANOVA analysis: in FST, both main effects were significant (CORT: F(1,25) = 15.61, *p* = 0.0006; CRS: F(1,25) = 9.96, *p* = 0.004), yet the CORT × CRS interaction was non-significant (F(1,25) = 1.83, *p* = 0.188), confirming additive rather than synergistic effects on behavioral despair. In striking contrast, hippocampal LC-MS analytes showed strong CORT × CRS interactions (e.g., hippocampal glutamate: F(1,8) = 47.87, *p* < 0.001; choline: F(1,8) = 56.21, *p* < 0.001; isoacetylcholine: F(1,8) = 103.39, *p* < 0.001), demonstrating genuine molecular-level synergy not captured by behavioral despair tests alone.

The OFT results further exemplify model-specific behavioral profiles. CORT administration induced selective reductions in center entries, center distance, and line crossings—potentially reflecting anxiety-related hypoactivity or reduced exploratory motivation—but neither CRS nor C+C groups did. This paradoxical finding aligns with Jeon et al. [17], who showed that CRS duration differentially affects anxiety- vs. depression-like behaviors, and Son et al. [18], who documented vendor-dependent variations in CRS-induced behavioral phenotypes. Importantly, the behavioral assays used here may not fully capture the anhedonic component of depression, and future studies should include sucrose preference testing, validated as a core depression endophenotype with higher translational validity than behavioral despair tests [6,30].

### 3.2. Complementary Neuroinflammatory Mechanisms

Inflammatory marker analysis revealed mechanistically informative patterns. CRS-containing groups showed greater IL-6 and TNF-α elevation than CORT alone, and Iba1 immunohistochemistry confirmed the most prominent microglial activation following CRS. This is consistent with evidence that physical stress activates the sympathoadrenal system and promotes microglial priming through catecholamine signaling [31], a pathway not engaged by exogenous CORT administration. The FKBP51-mediated regulation of hippocampal GAD65 expression provides a mechanistic link between stress-induced glucocorticoid receptor signaling and neuroinflammatory responses [32], and IL-17 signaling cumulatively induced by mild stress has been shown to drive depression-like behaviors through distinct neuroimmune pathways [33]. Recent evidence further demonstrates that targeted anti-inflammatory interventions can reverse stress-induced neuroinflammation: MgSO4 alleviates hippocampal neuroinflammation and blood–brain barrier damage in the CMS model [34], and imipramine reduces both peripheral and central nervous system inflammation along with behavioral improvements [35].

### 3.3. Serotonergic Dysregulation and the HTR1A Synergy

The most notable molecular finding in this study is the dramatic C+C-specific HTR1A (5-HT1A receptor) upregulation that significantly exceeded both individual treatment groups. This is particularly relevant to depression pathophysiology, as 5-HT1A functions as a major inhibitory autoreceptor on serotonergic neurons in the dorsal raphe nucleus, and its upregulation would suppress serotonin firing—precisely the mechanism that many antidepressant drugs target [36]. Peripheral serotonin levels have been proposed as biomarkers for depression diagnosis and treatment monitoring [37], and serum serotonin abnormalities are well-documented in depressed patients [38,39], supporting the translational relevance of serotonergic dysregulation in our model.

The simultaneous SERT upregulation across all groups further complicates serotonergic function, as it would enhance synaptic serotonin clearance. The combination of elevated HTR1A (decreased serotonin firing) and elevated SERT (enhanced serotonin reuptake) in the C+C group represents a particularly severe disruption of serotonergic transmission that neither model component could achieve alone. This dual mechanism mirrors the combination of glucocorticoid-dependent SERT upregulation described in chronic social defeat models [20] and stress-induced 5-HT1A autoreceptor sensitization documented in HPA axis dysregulation.

### 3.4. Neurotrophic Signaling and Neuronal Integrity

The reduction in BDNF, NGF, Trk-b, and DCX across experimental groups is consistent with the neurotrophic hypothesis of depression [40]. The C+C group showed particularly pronounced decreases in Trk-b and DCX, suggesting that the combined paradigm more effectively disrupts the neurotrophic signaling cascade from receptor to downstream neurogenesis. Zhang et al. [9] demonstrated that chronic 20 mg/kg CORT induces hyperactive neuronal autophagy that directly degrades BDNF protein, while CRS-mediated microglial activation can independently suppress neurotrophic factor production through inflammatory cytokine signaling [41]. The convergence of these two mechanisms in the C+C group may explain the enhanced suppression of the BDNF–Trk-b–neurogenesis pathway. Notably, Seo et al. [42] demonstrated that CRS-induced depression involves Fto-mediated regulation of CaMKII/CREB signaling in the hippocampus, suggesting an additional epigenetic mechanism by which restraint stress can suppress BDNF transcription independently of glucocorticoid receptor-mediated effects.

The Western blot analysis provides critical protein-level confirmation of these transcriptional changes. BDNF and NeuN protein reductions, particularly pronounced in the C+C group (68% and 61% reductions vs. Sham, respectively), validate the qRT-PCR and IHC findings and rule out post-transcriptional compensation. The phospho-protein analyses revealed a more nuanced pattern. The p-ERK/ERK ratio was most strongly reduced in C+C (54% reduction vs. Sham), consistent with combined paradigm-specific impairment of upstream ERK activation. Notably, however, the p-CREB/CREB ratio showed a different pattern: CRS produced the greatest p-CREB suppression (52% reduction), while CORT and C+C showed milder reductions (14% and 26%, respectively). This dissociation suggests that CRS preferentially suppresses CREB phosphorylation through ERK-independent pathways, consistent with Seo et al. [42], who demonstrated that CRS-induced depression involves Fto-mediated regulation of CaMKII/CREB signaling in the hippocampus. Since the ERK–CREB cascade is the principal intracellular pathway through which BDNF/TrkB receptor activation drives transcription of pro-survival and plasticity genes [40,42], the impairment of either node compromises the entire signaling axis. In the C+C group, the combination of severely reduced BDNF/Trk-b expression with maximal p-ERK suppression provides a mechanistic explanation for the selective neuronal loss (NeuN reduction) observed specifically in this group. The C+C-specific NeuN reduction represents one of the most consequential findings, indicating actual neuronal loss or degeneration beyond what either individual model produces. This is consistent with evidence that microglial glutaminase overexpression promotes excitotoxic synaptic damage during CRS [43], which when combined with CORT-induced autophagy and reduced neurotrophic support may exceed the threshold for neuronal survival.

### 3.5. Metabolomic Signatures and Peripheral Biomarkers

Serum metabolomics identified C+C-specific reductions in glutamine, dopamine, and adenosine—three markers each carrying distinct mechanistic implications. The serum adenosine reduction is particularly intriguing given the growing evidence for adenosine involvement in depression: Kaster et al. [44] demonstrated that adenosine administration produces antidepressant-like effects in mice through A1 and A2A receptors; Elgün et al. [45] showed decreased adenosine deaminase activity in depression; and Ortiz et al. [46] proposed the adenosinergic system as a novel therapeutic target based on its reciprocal relationship with monoaminergic neurotransmission. The serum adenosine decrease in our C+C model may therefore reflect the depletion of purinergic neuroprotective mechanisms under combined pharmacological-stress challenge, consistent with the view that adenosine receptors modulate both mood and anxiety through hippocampal and striatal circuits [47]. The specificity of these serum changes to the C+C group suggests they may serve as peripheral biomarker signatures distinguishing the combined HPA-stress depression phenotype.

### 3.6. Implications for Depression Model Selection

Our findings directly address Gururajan et al.’s [2] recommendation for multi-hit model approaches that better capture the multifactorial nature of human depression. The C+C model combines the reproducibility of pharmacological CORT administration with the ecological validity of physical stress, producing molecular and histological phenotypes spanning inflammatory, serotonergic, neurotrophic, cholinergic, glutamatergic, dopaminergic, and adenosinergic domains consistent with the multi-system pathology observed in clinical depression. Compared to other combined models, such as CRS + LPS [24] or chronic stress paradigms involving antidepressant administration through drinking water [48], the CORT+CRS paradigm offers the advantage of independent control over pharmacological and stress components, enabling mechanistic dissection of each contribution.

An important consideration in interpreting the present findings is the genetic background of C57BL/6N mice. The C57BL/6 lineage has been reported to exhibit a relatively pro-inflammatory immunological profile, including enhanced innate immune responsiveness and stress-related neuroinflammatory signaling, compared with some other inbred strains. Therefore, the pronounced Iba1 elevation and IL-6/TNF-α upregulation observed in the C+C group may have been partly amplified by the inflammatory predisposition of the C57BL/6N strain.

This strain-dependent effect is particularly relevant when comparing C57BL/6N mice with BALB/c mice, which are commonly described as having a relatively anti-inflammatory or Th2-skewed immune background. Under similar stress- or corticosterone-related experimental conditions, BALB/c mice may therefore show attenuated inflammatory responses compared with C57BL/6N mice, even when behavioral or HPA axis-related alterations are present. Thus, the inflammatory phenotype observed in the present C+C paradigm should not be interpreted as fully strain-independent. Future studies directly comparing C57BL/6N and BALB/c mice under the same CORT + CRS protocol would help clarify which inflammatory, neurotrophic, and behavioral changes represent core features of the combined paradigm.

Another important limitation is the exclusive use of male C57BL/6N mice in the present study. Because major depressive disorder is more prevalent in women, and sex differences in stress- and corticosterone-induced depression-like phenotypes are increasingly recognized, our findings and claims of model comprehensiveness should be interpreted as strictly applicable to male mice. Previous studies suggest that similar depressive-like outcomes may arise through sex-specific molecular mechanisms in stress-related models [49]. Therefore, future studies including female cohorts are required to determine whether the present behavioral, molecular, and histological findings can be generalized across sexes.

It should also be noted that human depression does not display the strongly polarized pro-inflammatory versus anti-inflammatory genetic backgrounds observed in inbred mouse strains. Rather, inflammatory profiles in major depressive disorder are heterogeneous across individuals and may vary according to sex, disease severity, stress exposure, metabolic status, and other clinical factors. Accordingly, the present findings should be translated cautiously, with recognition that the C57BL/6N background may emphasize the inflammatory component of the depression-like phenotype.

### 3.7. Limitations

Several limitations should be acknowledged. First, only male C57BL/6N mice were used, despite MDD being more prevalent in females and sex-specific molecular pathways being increasingly recognized; our claims of model comprehensiveness therefore apply strictly to males, and female cohorts are essential for future validation. Second, the behavioral battery (OFT, TST, FST) did not include anhedonia, social, or cognitive domains, and TST/FST immobility appeared to reach a ceiling across stress-exposed groups, limiting behavioral discrimination among CORT, CRS, and C+C paradigms. Third, immunohistochemical findings (Iba1, CD86, NeuN, BDNF) are presented qualitatively; descriptive terms such as “most prominent” should accordingly be interpreted as observations from representative images, not as quantitative comparisons. Because transcardial perfusion was not performed and brain tissues were rapidly harvested before immersion fixation, residual vascular or perivascular staining cannot be fully excluded, particularly for Iba1, which can label perivascular macrophages in addition to parenchymal microglia. Fourth, the Western blot densitometric quantification was performed on representative lane samples rather than all biological replicates, precluding formal statistical comparison at the protein level; these findings should be regarded as qualitative protein-level confirmation of the quantitative qRT-PCR data. Fifth, the use of a single C57BL/6N substrain (with a pro-inflammatory background) and modest group sizes (*n* = 7–8) may limit generalizability and statistical power for subtle effects. Sixth, the fixed four-week CORT/CRS duration precluded characterization of temporal dynamics. The present findings should therefore be regarded as an initial multi-domain characterization in male C57BL/6N mice, with broader generalization awaiting studies incorporating female cohorts, expanded behavioral assays, IHC quantification, and additional strains.

## 4. Materials and Methods

### 4.1. Animals

Male C57BL/6N mice (aged 7 weeks, weighing 35 g) were obtained from Dae Han Biolink Co., Ltd. (Eumseong, Republic of Korea). They were maintained under controlled temperature conditions (22 °C ± 2 °C) with a 12 h shifting light–dark cycle. The mice were housed five per cage with free access to food and water and provided with environmental enrichment such as nesting materials. Animals were monitored daily for general health, body weight, and behavior. All animals used in this study did not exhibit any conditions warranting euthanasia. All animal procedures were approved by the Institutional Animal Care and Use Committee of Dongguk University (IACUC2023-024-4).

### 4.2. Experimental Design and Groups

Male C57BL/6N mice were randomly assigned to four experimental groups: (1) Sham (*n* = 7); (2) Corticosterone (CORT; *n* = 7); (3) Chronic Restraint Stress (CRS; *n* = 7); and (4) Corticosterone + Chronic Restraint Stress (C+C; *n* = 8). To minimize the confounding effects of hormonal fluctuations on behavioral and molecular parameters, only male mice were utilized. Each animal was assigned a unique identification number, and investigators remained blinded to the group allocations during all behavioral testing and subsequent tissue analyses to ensure objectivity.

Corticosterone (CORT) was obtained from Tokyo Chemical Industry (TCI; Tokyo, Japan) and dissolved in a vehicle solution consisting of 0.1% dimethyl sulfoxide (DMSO; Sigma-Aldrich, St. Louis, MO, USA) and 2% polyoxyethylene sorbitan monooleate (Tween 80; Sigma-Aldrich). Mice in the CORT and C+C groups received daily subcutaneous (s.c.) injections of CORT at a dose of 20 mg/kg. The Sham group was administered daily s.c. injections of physiological saline as vehicle control.

For the chronic restraint stress procedure, mice in the CRS and C+C groups were individually placed into 50 mL conical tubes equipped with multiple ventilation holes. Restraint stress was applied for 3 h daily (from 13:00 to 16:00) over a period of 28 consecutive days. Immediately following the completion of the behavioral assessments, the mice were sacrificed. Tissues were then harvested for PCR, Western blot, liquid chromatography–mass spectrometry (LC/MS), and immunohistochemical (IHC) analyses.

### 4.3. Behavioral Assessments

Behavioral data were analyzed by an investigator blinded to group assignment. All behavioral tests were performed during the light phase, and the mice were allowed to habituate to the testing room for at least 1 h before each test. To eliminate olfactory cues, all apparatuses were thoroughly cleaned with 70% ethanol between trials.

#### 4.3.1. Open Field Test (OFT)

The OFT was conducted to evaluate spontaneous locomotor activity and anxiety-like behavior. Each mouse was gently placed in the center of a square open-field arena 40 × 40 × 40 cm and allowed to explore freely for 10 min. The animals’ movements were recorded and analyzed using the ANY-maze video tracking system (Stoelting Co., Wood Dale, IL, USA). The arena was conceptually divided into a central zone and a peripheral zone. The total distance traveled (cm) and the entries in the center zone were measured to assess general locomotion and anxiety levels, respectively.

#### 4.3.2. Tail Suspension Test (TST)

The TST was performed to assess depressive-like behavior. Mice were individually suspended approximately 50 cm above the floor using adhesive tape placed about 1 cm from the tip of the tail. To prevent mice from observing each other, they were visually isolated during the test. The total duration of the test was 6 min. The immobility time was manually scored during the last 4 min of the test by an investigator blinded to the experimental groups. Mice were considered immobile only when they hung passively and remained completely motionless.

#### 4.3.3. Forced Swim Test (FST)

The FST was also used to evaluate depressive-like behavior. Each mouse was placed individually in a transparent cylindrical glass tank (height, 30 cm; diameter, 20 cm) filled with water (24 ± 1 °C) to a depth of 15 cm, ensuring that the mice could not touch the bottom with their hind paws or tails. The test was conducted for a total of 6 min. Following an initial 2-min habituation period, the immobility time was manually recorded during the subsequent 4 min by a blinded investigator. A mouse was judged to be immobile when it ceased struggling and remained floating motionless, making only the minimal movements necessary to keep its head above water. After the test, the mice were immediately removed, dried with a paper towel, and returned to their home cages.

### 4.4. Reverse Transcription Followed by Quantitative PCR (qRT-PCR)

Bilateral hippocampi were harvested from the mice for sample preparation. Total RNA isolation from the cells was performed using the RNAiso Plus reagent (1 mL/sample; Takara Bio, Shiga, Japan) according to the manufacturer’s protocol. Following the addition of 200 μL of chloroform (Sigma, Tokyo, Japan) to the homogenate, the mixture was incubated for 3 min at room temperature. The samples were then centrifuged at 12,000× *g* for 15 min at 4 °C, and the upper aqueous phase was carefully transferred to a fresh tube. To precipitate the RNA, 500 μL of isopropanol was added to the aqueous layer, followed by a 10-min incubation at room temperature. After centrifugation at 12,000× *g* for 10 min, the resulting pellets were washed with 1 mL of 75% ethanol. A subsequent centrifugation step at 7500× *g* for 5 min at 4 °C was conducted, after which the supernatant was discarded, and the RNA pellets were air-dried at room temperature. The dried pellets were resuspended in 20 μL of RNase-free water and heated at 65 °C for 10 min using a heating block. RNA concentration and purity were evaluated utilizing a NanoDrop spectrophotometer (Thermo Fisher Scientific, Waltham, MA, USA). First-strand cDNA was synthesized from the extracted total RNA using a Reverse Transcription Master Mix (Dynebio, Seongnam, Republic of Korea). Quantitative real-time PCR (qPCR) assays were carried out on a StepOnePlus™ Real-Time PCR System (Applied Biosystems, Thermo Fisher Scientific, Waltham, MA, USA) utilizing TB Green^®^ Premix Ex Taq™ (Takara Bio, Shiga, Japan). Relative gene expression variations were calculated applying the comparative threshold cycle (ΔΔCT) method. The specific primer sequences used in this study are detailed in Table 1.

### 4.5. Western Blot Analysis

Bilateral mouse hippocampi were dissected and homogenized in a sample lysis buffer comprising 0.25 M Tris-HCl (pH 6.8), 4% SDS, 40% glycerol, 0.05% bromophenol blue, and 10% β-mercaptoethanol. The tissue homogenates were centrifuged at 12,000× *g* for 15 min at 4 °C, and the supernatants were collected. Total protein concentrations were determined via the bicinchoninic acid (BCA) assay, utilizing bovine serum albumin (BSA) to generate a standard curve for absorbance-based calculations. For SDS-PAGE, protein samples were resolved on 10% SDS–polyacrylamide gels and electrophoresed at 90 V for 120 min. With an average protein concentration of approximately 6 µg/µL, 4.9 µL of the tissue lysate was combined with the loading buffer to reach a final volume of 20 µL per well, ensuring a consistent loading amount of 30 µg of protein per lane. All immunoblotting assays were conducted using identical protein extracts from the same sample cohort, with equal amounts loaded onto separate gels for each target protein. The separated proteins were subsequently transferred onto polyvinylidene difluoride (PVDF) membranes. Non-specific binding was blocked by incubating the membranes in 5% non-fat skim milk at room temperature for 1 h. After three 10-min washes in Tris-buffered saline containing 0.1% Tween 20 (TBS-T), the membranes were probed with primary antibodies for 1 h. Following additional TBS-T washes, the blots were incubated with the appropriate secondary antibodies, diluted in 5% skim milk, for 2 h at room temperature. Protein bands were visualized using an enhanced chemiluminescence (ECL) detection system, and images were captured via a ChemiDoc XRS+ Imaging System (Bio-Rad, Hercules, CA, USA). Details of the primary and secondary antibodies used for Western blot analysis, including dilution ratios, manufacturers, and catalog numbers, are provided in Table 2.

### 4.6. Immunohistochemical Analysis

Mouse brain tissues were harvested and fixed overnight at 4 °C in a 4% paraformaldehyde (PFA) solution. The fixed specimens were subsequently dehydrated through a graded ethanol series and embedded in paraffin blocks. The paraffin-embedded tissues were sectioned at a thickness of 4 μm, mounted onto glass slides, and air-dried at room temperature. The slides were then baked at 65 °C for 2 h. Following deparaffinization and rehydration, endogenous peroxidase activity was quenched by immersing the slides in hydrogen peroxide for 10 min at room temperature, followed by rinsing in PBS-T (phosphate-buffered saline containing 0.1% Tween-20). Tissue sections were then incubated overnight at 4 °C with the respective primary antibodies. Post-incubation, the slides were exposed to an HRP-conjugated anti-rabbit/mouse secondary antibody (Agilent, #K5007, Santa Clara, CA, USA) for 30 min at room temperature. Colorimetric development was achieved using 3,3′-diaminobenzidine (DAB; Agilent Dako, #K5007). The reaction proceeded for 3 min for most markers, while an optimized 4-min incubation was utilized for BDNF to obtain robust signals. After washing with PBS-T, the sections were counterstained with Mayer’s Hematoxylin (MUTO PURE CHEMICALS, #30002, Tokyo, Japan) for 4 min. Histological images were acquired using a light microscope. Table 3 summarizes the dilution ratios, sources, and catalog numbers of the antibodies utilized for immunohistochemistry.

### 4.7. LC-MS/MS Analysis

Concentrations of neurotransmitters and metabolites in hippocampal tissue and serum were quantified by ultra-high-performance liquid chromatography coupled with tandem mass spectrometry (UHPLC-MS/MS), following an established bioanalytical protocol validated according to the Korean Ministry of Food and Drug Safety (MFDS) Guideline on Bioanalytical Method Validation (2013) and the U.S. FDA Guidance for Industry: Bioanalytical Method Validation (2001). The target analytes assessed in this study were glutamate (Glu), glutamine (Gln), acetylcholine (ACh), isoacetylcholine (Iso-ACh), choline (Ch), dopamine (DA), and adenosine (ADE), selected based on their established roles in depression pathophysiology.

#### 4.7.1. Sample Preparation

Bilateral hippocampal tissue was weighed and homogenized in 10× volume (*w*/*v*) of phosphate-buffered saline (PBS) using a tissue homogenizer, then stored at −70 °C until analysis. Serum samples were prepared by centrifugation of whole blood at 3000 rpm for 15 min at 4 °C. For analysis, 50 μL aliquots of hippocampal homogenate or serum were processed by protein precipitation. Samples were spiked with internal standard, mixed with the diluent solution (water containing 0.25 mM ascorbic acid and 0.1 M perchloric acid) to stabilize catecholamines and prevent oxidation, and centrifuged. The resulting supernatants were transferred to autosampler vials for injection.

#### 4.7.2. Chromatographic Conditions

Chromatographic separation was performed on a Vanquish UHPLC system (Thermo Fisher Scientific, Waltham, MA, USA) equipped with a Kinetex^®^ Biphenyl column (2.1 × 100 mm, 1.7 μm; Phenomenex, Torrance, CA, USA) maintained at 30 °C. The mobile phase consisted of (A) distilled water with 0.1% (*v*/*v*) formic acid and (B) acetonitrile/methanol (1:1, *v*/*v*), delivered at a flow rate of 0.20 mL/min using gradient elution. The injection volume was 2.0 μL.

#### 4.7.3. Mass Spectrometric Detection

Mass spectrometric detection was performed on a TSQ Altis triple-quadrupole tandem mass spectrometer (Thermo Fisher Scientific) equipped with a heated electrospray ionization (H-ESI) source operating in both positive and negative ionization modes. Quantification was performed in multiple reaction monitoring (MRM) mode using the following analyte-specific transitions: Glu, *m*/*z* 148.038 → 84.155 (retention time, 1.06 min); Gln, *m*/*z* 147.125 → 129.982 (1.16 min); ACh, *m*/*z* 146.125 → 87.125 (2.27 min); Iso-ACh, *m*/*z* 146.175 → 60.137 (2.04 min); Ch, *m*/*z* 104.125 → 60.155 (1.28 min); DA, *m*/*z* 154.125 → 137.125 (2.17 min); and ADE, *m*/*z* 268.088 → 136.125 (2.95 min).

#### 4.7.4. Calibration and Quality Control

Calibration standards were prepared in the diluent solution by serial dilution of a mixed working standard. Calibration ranges were 100–10,000 ng/mL for Gln, Ch, and Glu, and 1–100 ng/mL for ACh, Iso-ACh, DA, and ADE. Six-point calibration curves were generated for each analyte using quadratic regression with 1/x^2^ weighting (Xcalibur software, version 4.1.31.9; Thermo Fisher Scientific). Quality control (QC) samples were prepared at four concentrations (LLOQ, low, medium, and high QC) and analyzed in duplicate within each batch. Acceptance criteria for calibration standards and QC samples were ±15% deviation from nominal concentration (±20% at LLOQ), in accordance with international bioanalytical validation guidelines.

#### 4.7.5. Concentration Calculation

Final analyte concentrations in hippocampal homogenate samples were back-calculated from the calibration curves and corrected for the 10-fold dilution factor introduced during tissue homogenization to express results as ng per gram of wet tissue. Serum analyte concentrations were reported in ng/mL. All chromatographic peaks were manually inspected, and only peaks with signal-to-noise ratios ≥10:1 and accurate retention time were accepted for quantification.

### 4.8. Statistical Analysis

All empirical data are expressed as the mean ± standard error of the mean (SEM). To determine statistically significant differences among experimental groups, a one-way analysis of variance (ANOVA) was performed, followed by Tukey’s post hoc test for multiple comparisons. A *p*-value of less than 0.05 was considered the threshold for statistical significance. Significance levels are denoted as follows: * *p* < 0.05, *p* < 0.01, *** *p* < 0.005, and **** *p* < 0.001. In addition, a two-way ANOVA (2 × 2 factorial design with CORT and CRS as independent factors) was conducted for each endpoint to evaluate main effects of each stressor and the CORT × CRS interaction term, enabling identification of synergistic versus additive effects. F statistics, degrees of freedom, *p* values, and interaction terms for all endpoints are reported in the Supplementary Statistics Tables. All statistical analyses were performed using GraphPad Prism (version 10.6.1).

## 5. Conclusions

This study demonstrates that the combined CORT + CRS paradigm produces the most comprehensive depression-related molecular and histological profile compared to either approach alone, while behavioral despair measures (TST, FST) showed comparable elevation across all stress-exposed groups, suggesting a behavioral ceiling effect that limits behavioral discrimination among CORT, CRS, and C+C paradigms. While behavioral despair measures showed uniform elevation without synergistic effects, molecular analyses revealed C+C-specific advantages including (1) synergistic HTR1A upregulation indicating enhanced serotonergic inhibition; (2) the most pronounced disruption of neurotrophic signaling across the BDNF–Trk-b–DCX cascade; (3) unique serum dopamine, glutamine, and adenosine reductions reflecting multi-system metabolic disruption; (4) the most severe suppression of upstream ERK phosphorylation (p-ERK/ERK ratio reduced 54% vs. Sham), with parallel impairment of downstream p-CREB activation observed across all experimental paradigms; and (5) selective neuronal loss (NeuN reduction of 61%) not observed with either individual treatment. The dissociation between behavioral and molecular outcomes reinforces the critical importance of multi-domain assessment in depression model validation and cautions against reliance on behavioral tests alone for evaluating model adequacy. We propose the combined CORT + CRS model as a comprehensive preclinical paradigm at the molecular and histological levels, recognizing that behavioral validity is restricted to specific domains. Future studies incorporating anhedonia measures (e.g., sucrose preference), social behavior tests, cognitive assessments, and female cohorts will be required to fully validate the model’s behavioral utility and translational relevance to human depression.

## Figures and Tables

**Table 1:** List of primer sequences.

Gene	Forward (5′–3′)	Reverse (5′–3′)
*Mouse β* *-actin*	AGGCCAACCGTGAAAAGATG	TGGCGTGAGGGAGAGCATAG
*Mouse TNF-* *α*	CACTCACAAACCACCAAGTG	GAGTAGACAAGGTACAACCC
*Mouse IL-6*	CTG CAA GAG ACT TCCATC CAG TT	GAA GTA GGG AAG GCC GTG G
*Mouse SERT*	GTTGATGCTGCGGCTCAGATCT	GAAGCTCGTCATGCAGTTCACC
*Mouse HTR1A*	TAC TCC ACT TTC GGC GCT TT	CTG CAA AAA GCA CTG TCC CC
*Mouse CRH*	CTG ATC CGC ATG GGT GAA GA	GGA AAA AGT TAG CCG CAG CC
*Mouse BDNF*	GAC CCT TCT TAT CGC TGG G	AGC AAT CAG TTT GTT CGG CTC
*Mouse NGF*	GTTTTGCCAAGGACGCAGCTTTC	GTTCTGCCTGTACGCCGATCAA
*Mouse Trk B*	CCA CGG ATG TTG CTG ACC AAA G	GCC AAA CTT GGA ATG TCT CGC C
*Mouse DCX*	CTGACTCAGGTAACGACCAAGAC	TTCCAGGGCTTGTGGGTGTAGA

**Table 2:** List of primary and secondary antibodies used for Western blot analysis. Table includes antibody names, dilution ratios, sources (companies), and catalog numbers.

Antibody	Dilution	Company	Catalog Number
anti-β-actin	1:5000	Abcam	ab8227
anti-Iba-1	1:1000	GeneTex	GTX635363
anti-BDNF	1:1000	GeneTex	GTX132621
anti-NeuN	1:1000	abcam	ab279296
anti-ERK	1:1000	Cell Signaling	9102S
anti-p-ERK	1:1000	Cell Signaling	9101S
anti-CREB	1:1000	Cell Signaling	9197S
anti-p-CREB	1:1000	Cell Signaling	9198S
Goat anti-mouse IgG HRP	1:3000	Abcam	ab6728
Goat anti-rabbit IgG HRP	1:3000	Abcam	ab6721

**Table 3:** List of primary and secondary antibodies used for immunohistochemistry analysis. Table includes antibody names, dilution ratios, sources (companies), and catalog numbers.

Antibody	Dilution	Company	Catalog Number
anti-CD86	1:100	GeneTex	GTX34569
anti-Iba-1	1:400	GeneTex	GTX635363
anti-NeuN	1:1000	abcam	ab279296
anti-BDNF	1:200	GeneTex	GTX132621
anti-rabbit/mouse HRP	-	Agilent	K5007

## Data Availability

The original contributions presented in this study are included in the article. Further inquiries can be directed to the corresponding author.

## Supplementary Materials

The following supporting information can be downloaded at: https://www.mdpi.com/article/10.3390/ijms27146277/s1.

## Abbreviations

The following abbreviations are used in this manuscript:

ACh	Acetylcholine
ADE	Adenosine
ANOVA	Analysis of variance
BDNF	Brain-derived neurotrophic factor
Ch	Choline
CORT	Corticosterone
CRH	Corticotropin-releasing hormone
CRS	Chronic restraint stress
DA	Dopamine
DCX	Doublecortin
ERK	Extracellular signal-regulated kinase
FST	Forced swim test
Gln	Glutamine
Glu	Glutamate
HPA	Hypothalamic–pituitary–adrenal
HTR1A	5-Hydroxytryptamine receptor 1A
IACUC	Institutional Animal Care and Use Committee
Iba1	Ionized calcium-binding adapter molecule 1
IHC	Immunohistochemistry
IL-6	Interleukin 6
Iso-ACh	Isoacetylcholine
LC-MS/MS	Liquid chromatography-tandem mass spectrometry
MDD	Major depressive disorder
MRM	Multiple reaction monitoring
NeuN	Neuronal nuclear protein
NGF	Nerve growth factor
OFT	Open field test
p-CREB	Phosphorylated cyclic AMP response element-binding protein
p-ERK	Phosphorylated ERK
qRT-PCR	Quantitative reverse-transcription polymerase chain reaction
SEM	Standard error of the mean
SERT	Serotonin transporter
TNF-α	Tumor necrosis factor alpha
Trk-b	Tropomyosin receptor kinase B
TST	Tail suspension test
UHPLC	Ultra-high-performance liquid chromatography
