# Supplementary material for: Comparative Evaluation of Corticosterone Administration, Chronic Restraint Stress, and Their Combination for Depression-like Behavioral and Molecular Alterations in Mice: A Multi-Domain Assessment
Source: Int J Mol Sci. 2026 Jul 14;27(14):6277. doi: 10.3390/ijms27146277 (PMC13410486; doi:10.3390/ijms27146277)
Supplement: Supplementary file 1 [file ijms-27-06277-s001.zip › Table_S1_Primer_Sequences.pdf]

Supplementary Materials

Manuscript: Comparative Evaluation of Corticosterone Administration, Chronic Restraint Stress, and Their Combination for Depression Model Formation in Mice

Table S1. Primer Sequences Used for Quantitative Real-Time PCR Analysis

Forward and reverse primer sequences (5′→3′) for the ten murine genes analyzed by qRT-PCR in this study. β-actin was used as the reference gene for normalization. All primers were designed for use with TB Green® Premix Ex Taq™ on the StepOnePlus™ Real-Time PCR System.

No.	Gene	Forward primer (5′→3′)	Reverse primer (5′→3′)	Functional category
1	***β-actin***	AGGCCAACCGTGAAAAGATG	TGGCGTGAGGGAGAGCATAG	*Reference (housekeeping)*
2	***TNF-α***	CACTCACAAACCACCAAGTG	GAGTAGACAAGGTACAACCC	*Inflammation*
3	***IL-6***	CTGCAAGAGACTTCCATCCAGTT	GAAGTAGGGAAGGCCGTGG	*Inflammation*
4	***SERT***	GTTGATGCTGCGGCTCAGATCT	GAAGCTCGTCATGCAGTTCACC	*Serotonergic*
5	***HTR1A***	TACTCCACTTTCGGCGCTTT	CTGCAAAAAGCACTGTCCCC	*Serotonergic*
6	***CRH***	CTGATCCGCATGGGTGAAGA	GGAAAAAGTTAGCCGCAGCC	*HPA axis*
7	***BDNF***	GACCCTTCTTATCGCTGGG	AGCAATCAGTTTGTTCGGCTC	*Neurotrophic*
8	***NGF***	GTTTTGCCAAGGACGCAGCTTTC	GTTCTGCCTGTACGCCGATCAA	*Neurotrophic*
9	***Trk-b***	CCACGGATGTTGCTGACCAAAG	GCCAAACTTGGAATGTCTCGCC	*Neurotrophic (BDNF receptor)*
10	***DCX***	CTGACTCAGGTAACGACCAAGAC	TTCCAGGGCTTGTGGGTGTAGA	*Neurogenesis*

Notes:

- Total RNA was extracted from bilateral hippocampal tissue using RNAiso Plus reagent (Takara Bio, Shiga, Japan) according to the manufacturer's protocol.
- First-strand cDNA was synthesized using a Reverse Transcription Master Mix (Dynebio, Republic of Korea).
- qRT-PCR was performed on a StepOnePlus™ Real-Time PCR System (Applied Biosystems, Thermo Fisher Scientific, Waltham, MA, USA) using TB Green® Premix Ex Taq™ (Takara Bio).
- Cycling conditions: initial denaturation at 95 °C for 30 s, followed by 40 cycles of 95 °C for 5 s and 60 °C for 30 s.
- Relative gene expression was calculated using the comparative threshold cycle ($2^{-\Delta\Delta CT}$) method, with β-actin as the endogenous reference gene.
- All primer pairs were validated for efficiency (90–110%) and amplification specificity (single melt curve peak) prior to experimental use.

Abbreviations: *BDNF, brain-derived neurotrophic factor; CRH, corticotropin-releasing hormone; DCX, doublecortin; HTR1A, 5-hydroxytryptamine receptor 1A (serotonin receptor 1A); IL-6, interleukin-6; NGF, nerve growth factor; SERT, serotonin transporter (also known as SLC6A4); TNF-α, tumor necrosis factor alpha; Trk-b, tropomyosin receptor kinase B (BDNF receptor, also known as TrkB or NTRK2).*
