# Supplementary figures and images for: Comparative Evaluation of Corticosterone Administration, Chronic Restraint Stress, and Their Combination for Depression-like Behavioral and Molecular Alterations in Mice: A Multi-Domain Assessment
Source: Int J Mol Sci. 2026 Jul 14;27(14):6277. doi: 10.3390/ijms27146277 (PMC13410486; doi:10.3390/ijms27146277)

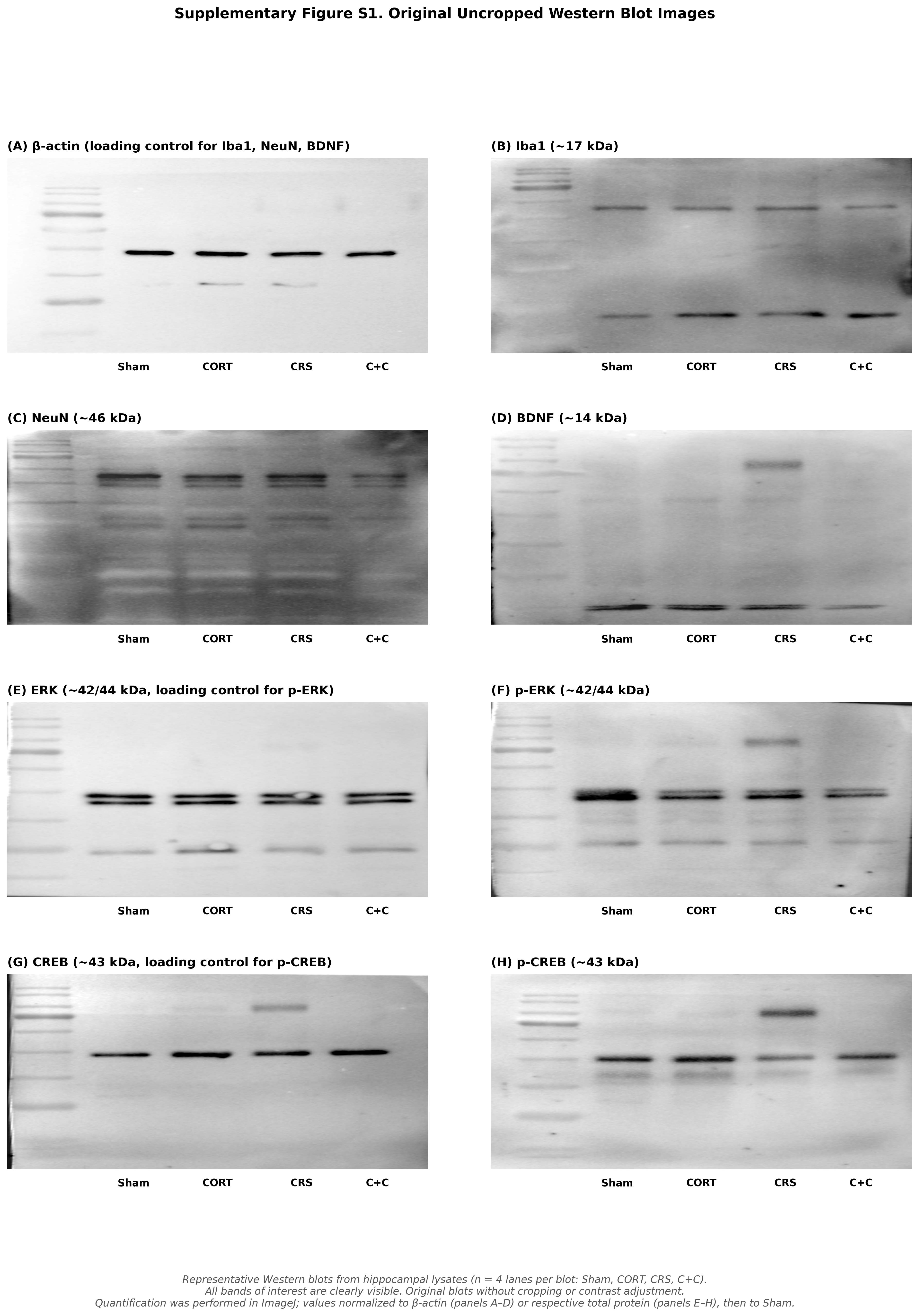

Supplement: Supplementary file 1 [file ijms-27-06277-s001.zip › Supplementary_Figure_S1.tiff]
